# Potential Involvement of Redox and Inflammatory Signalling in the Antihyperglycemic and Antioxidant Effects of Harmaline in Male Mice With Type 2 Diabetes

**DOI:** 10.1002/edm2.70304

**Published:** 2026-08-10

**Authors:** Farima Malekinia, Akram Ahangarpour, Khojasteh Hoseinynejad, Seyyed Ali Mard, Maryam Radan, Fereshteh Nejaddehbashi

**Affiliations:** ^1^ Student Research Committee Ahvaz Jundishapur University of Medical Sciences Ahvaz Iran; ^2^ Diabetes Research Center, Health Research Institute Ahvaz Jundishapur University of Medical Sciences Ahvaz Iran; ^3^ Persian Gulf Physiology Research Center, Medical Basic Sciences Research Institute Ahvaz Jundishapur University of Medical Sciences Ahvaz Iran; ^4^ Cellular and Molecular Research Center, Medical Basic Sciences Research Institute Ahvaz Jundishapur University of Medical Sciences Ahvaz Iran

**Keywords:** harmaline, inflammation, male mice, oxidative stress, type 2 diabetes

## Abstract

**Introduction:**

Type 2 diabetes mellitus (T2DM) is strongly associated with oxidative stress and inflammation. Harmaline (HAR), an alkaloid with antioxidant and anti‐inflammatory properties, has been suggested to possess antidiabetic potential. This study investigated the effects of HAR in experimental T2DM and its association with changes in the nuclear factor erythroid 2‐related factor 2 (Nrf2) and nuclear factor kappa‐B (NF‐κB) pathways.

**Methods:**

In vitro experiments were performed using mouse pancreatic cells cultured under different glucose concentrations to evaluate insulin secretion and antioxidant status. For the in vivo study, T2DM was induced in mice by nicotinamide (120 mg/kg) and streptozotocin (65 mg/kg). Fasting blood glucose (FBG) was measured 1 week after induction. Forty‐eight adult male NMRI mice were randomly assigned to four groups: normal control (NC), diabetic control (DC), HAR‐treated (30 mg/kg), and metformin‐treated (150 mg/kg). Treatments were administered once daily for 10 days.

**Results:**

HAR enhanced insulin secretion and antioxidant capacity while reducing lipid peroxidation in vitro. In diabetic mice, HAR significantly reduced FBG, improved the lipid profile, and restored pancreatic antioxidant status. These effects were accompanied by increased expression of miR‐200a, Nrf2, and NAD(P)H: quinone oxidoreductase 1 and reduced Keap1 expression. In addition, HAR increased miR‐125b expression and reduced the expression of NF‐κB, TNF‐α, IL‐1β, and IL‐6. Decreased changes in RAS‐related markers were also observed after HAR treatment.

**Conclusion:**

HAR improved glycemic control and pancreatic antioxidant status and reduced inflammatory responses in experimental T2DM. These beneficial effects were associated with modulation of Nrf2, NF‐κB, and RAS‐related signalling pathways, suggesting their potential involvement in the observed protective effects. Further studies are needed to establish the underlying mechanisms and evaluate the long‐term efficacy and safety of HAR.

## Graphical abstract

HAR may improve T2DM by enhancing antioxidant defences through Nrf2 activation while suppressing inflammation and RAS activity, supporting its capability to treat diabetes.

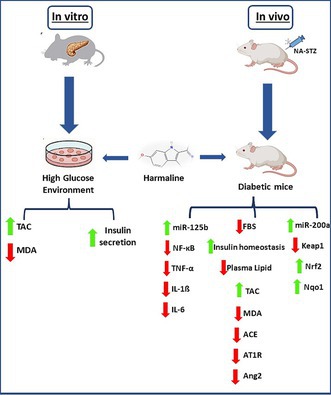

## 1 Introduction

The intricate pathogenic pathways of Type 2 diabetes mellitus (T2DM), a multifactorial disease, disrupt the regulation of endocrine function and metabolism [1, 2]. Pathogenesis of T2DM is mainly driven by impaired insulin secretion due to pancreatic β‐cell damage and insulin resistance [3, 4]. Two important factors, oxidative stress and inflammation, if overactive or prolonged, promote T2DM and its complications [5]. The high rate of ROS production and insufficient antioxidant capacity make pancreatic cells vulnerable to oxidative damage [6]. Consequently, the development of these two factors is closely related to pancreatic beta cell damage in type 2 diabetes [7]. Evidence from experimental studies indicates that antioxidant therapy can attenuate oxidative stress in diabetic mice, resulting in increased insulin content and enhanced insulin mRNA expression [8, 9].

Nuclear factor erythroid‐related 2 (Nrf2), a regulator of cytoprotective response [10], plays a vital role in cellular defence mechanisms by regulating cellular redox balance through increased expression of antioxidant enzymes [11]. The Kelch‐like ECH‐associated protein 1 (Keap1) negatively regulates Nrf2's activity [12]. MicroRNAs (miR), short non‐coding RNAs, play a vital role in numerous biological functions, notably in controlling gene expression [13]. Evidence suggests that miR‐200a targets Keap1 mRNA and degrades it. This activates the Nrf2 signalling pathway [14]. Considering the points mentioned, the activation of Nrf2 is useful in controlling and treating diabetes, but also in preventing its complications [15].

Nuclear factor kappa beta (NF‐κB) activates inflammatory genes by activating transcription [16]. Increased levels of tumour necrosis factor alpha (TNF‐α), interleukin‐1 beta (IL‐1β), and interleukin‐6 (IL‐6), which are inflammatory cytokines, are attributed to increased NF‐κB expression in T2DM [17]. miR‐125b contributes to suppressing pro‐inflammatory cytokine production by inversely correlating with NF‐κB expression [18]. When the renin‐angiotensin system (RAS) is more active, it leads to impaired insulin secretion from pancreatic beta cells due to reduced pancreatic blood flow, islet fibrosis, oxidative stress, and inflammation. It also causes insulin resistance by impairing skeletal muscle and adipose tissue function [19].

The nicotinamide (NA)‐streptozotocin (STZ) model is extensively used to induce T2DM and test antidiabetic agents. The combination of these two chemicals slows down the damaging effect of STZ on all pancreatic beta cells, allowing the remaining functional cells to respond to glucose stimulation [20].

Since ancient times, nature has been a major source of medicinal agents, and herbal products have contributed to disease treatment and drug discovery [21]. Metformin remains the first‐line oral therapy for T2DM [22]. Although highly effective during early treatment, its efficacy may decline over time [23]. It can cause adverse effects, including gastrointestinal disorders [24], lactic acidosis [25], hepatotoxicity [26], and acute pancreatitis [27]. Therefore, the development of novel herbal or natural antidiabetic agents that act through mechanisms involved in glucose homeostasis and pancreatic function remains an important therapeutic goal. Harmaline (HAR) is an alkaloid from *Peganum harmala* L. extract that has antioxidant [28], anti‐inflammatory, against liver cirrhosis [29], anticancer [30], antidepressant [31], and anxiolytic properties [32]. Harmaline has been able to reduce kidney inflammation by suppressing oxidative stress [33].

According to these properties, this research was performed to assess the potential antidiabetic properties of harmaline in male mice suffering from T2DM mellitus brought on by nicotinamide and streptozotocin, concentrating on its influence on the Signalling pathways of Nrf2 and NF‐kB and the RAS system, considering that the antidiabetic effects of harmaline have not been explored before.

## 2 Materials and Methods

### 2.1 In Vitro Experiments

#### 2.1.1 Isolation of Langerhans' Islets and Insulin Secretion in Various Glucose Concentrations

Pancreatic islets were isolated as previously described [34, 35]. Briefly, once deeply anaesthetised with ketamine (100 mg/kg) and xylazine (10 mg/kg), the pancreas was excised and kept in Krebs–bicarbonate buffer (pH 7.4; 115 mM NaCl, 5 mM KCl, 2.56 mM CaCl_2_, 1 mM MgCl_2_, 10 mM NaHCO_3_, and 15 mM HEPES). The tissue was minced into small fragments and digested with collagenase P at 37°C. Digestion was terminated by adding cold buffer, followed by centrifugation, and Langerhans' islets were manually isolated under a stereomicroscope.

For insulin secretion studies, isolated islets were incubated in Krebs–bicarbonate buffer containing varying glucose concentrations (2.8, 5.6, or 16.7 mM) [36]. Harmaline (5, 10, or 15 μM) was added at the start of the incubation. After 45 min at 37°C, samples were centrifuged (100 × g, 5 min), and until insulin measurement, the supernatants were gathered and preserved at −70°C. Each experimental condition was performed in eight replicates, requiring a total of 32 mice.

#### 2.1.2 Study Grouping

This part of the study was conducted on 15 groups, with six islets of Langerhans isolated from four mice in each group. This procedure was repeated eight times, requiring a total of 32 mice.

According to the above, the grouping of the present study is as follows:

- Group 2.8: receiving a glucose concentration of 2.8 mM.
- Group 5.6: receiving a glucose concentration of 5.6 mM.
- Group 16.7: receiving a glucose concentration of 16.7 mM.
- Group 2.8 + 5HAR: receiving harmaline 5 μM with glucose concentration of 2.8 mM.
- Group 5.6 + 5HAR: receiving harmaline 5 μM with glucose concentration of 5.6 mM.
- Group 16.7 + 5HAR: receiving harmaline 5 μM with glucose concentration of 16.7 mM.
- Group 2.8 + 10HAR: receiving harmaline 10 μM with glucose concentration of 2.8 mM.
- Group 5.6 + 10HAR: receiving harmaline 10 μM with glucose concentration of 5.6 mM.
- Group 16.7 + + 10HAR: receiving harmaline 10 μM with glucose concentration of 16.7 mM.
- Group 2.8 + 15HAR: receiving harmaline 15 μM with glucose concentration 2.8 mM.
- Group 5.6 + 15HAR: receiving 15 μM harmaline with glucose concentration 5.6 mM.
- Group 16.7 + 15HAR: receiving 15 μM harmaline with glucose concentration 16.7 mM.
- Group 2.8 + G: receiving 10 μM glibenclamide with glucose concentration 2.8 mM (positive control).
- Group 5.6 + G: receiving 10 μM glibenclamide with glucose concentration 5.6 mM (positive control).
- Group 16.7 + G: receiving 10 μM glibenclamide with glucose concentration 16.7 mM (positive control).

#### 2.1.3 Insulin Measurements

To assess the amount of insulin, the liquid samples collected from the extracted islets of Langerhans, which contain the hormone, are taken out of the freezer and allowed to reach room temperature. Once they are thawed, the amount of insulin in each sample is measured using the ELISA technique with a specialized insulin measurement kit (Diametra, Iran).

#### 2.1.4 Assessment of Lipid Peroxidation and Antioxidant

To assess the extent of lipid peroxidation (MDA level) and TAC in isolated islets of Langerhans, the enzyme‐linked immunosorbent assay (ELISA) technique was employed as per the kit manufacturer's instructions (ZellBio, Germany).

### 2.2 In Vivo Experiments

#### 2.2.1 Medications and Substances

The following drugs and chemicals were obtained from Sigma‐Aldrich, a company based in the United States: Nicotinamide, streptozotocin, harmaline, metformin (MET), and Bradford reagent. We acquired from Merck, which is based in Germany, sodium citrate dihydrate, citric acid, sodium hydroxide, hydrochloric acid, and bovine serum albumin. Ketamine and xylazine were obtained from Alfasan, Netherlands.

#### 2.2.2 Animal Models and Experimental Design

The sample size was determined based on commonly accepted practices for exploratory preclinical animal studies and was consistent with previous investigations employing similar experimental designs [37, 38]. A total of 48 male NMRI mice (6–8 weeks old, 20–30 g) were obtained from the Animal Experiment Center of Ahvaz Jundishapur University of Medical Sciences. All experimental procedures were approved by the institutional Ethics Committee of Ahvaz Jundishapur University of Medical Sciences, Ahvaz, Iran (Ethical Approval number IR.AJUMS.AEC.1403.012). Animals were housed under standard conditions (12 h light/12 h dark cycle) with free access to food and water and allowed to acclimatize for 7 days before the study. In addition, randomization and blinding procedures were implemented during the study to minimize selection and observer bias. T2DM was induced in 36 mice using the nicotinamide (NA)/streptozotocin (STZ) model. Following an 8‐h fast, mice received an intraperitoneal injection of NA (120 mg/kg), followed 15 min later by STZ (65 mg/kg, into a fresh citrate buffer at pH 4.5) [39]. The remaining 12 mice received vehicle only and served as the normal control group. One week after STZ administration, fasting blood glucose (FBG) was measured from tail‐vein blood samples using a glucometer. Mice with FBG ≥ 200 mg/dL and diabetic symptoms, including polyuria, were considered diabetic and randomly allocated to experimental groups. One week after diabetes confirmation, treatment protocols were initiated according to group assignment:

1. 12 mice were given normal saline by gavage for ten successive days and were classified as the normal control (NC) group.
2. 12 diabetic mice were also given normal saline by gavage for ten successive days and were classified as the diabetic control (DC) group.
3. 12 diabetic mice received harmaline at a dose of 30 mg per kilogram [40] of body weight daily via gavage for ten consecutive days and were classified as the harmaline (HAR) group.
4. 12 diabetic mice were administered metformin at a dose of 150 mg per kilogram of body weight daily through gavage for ten consecutive days. They were classified as the metformin (MET) group for positive control.

During the experimental period, no mortality or overt signs of systemic toxicity were observed in any of the experimental groups. Once the 10‐day treatment was finished, FBG was measured. Mice were euthanized by intraperitoneal administration of ketamine (100 mg/kg) and xylazine (10 mg/kg). Cardiac puncture was used to collect blood samples, which were centrifuged (3000 rpm, 10 min), and plasma was separated and stored at −20°C until analysis. The pancreas was quickly removed, rinsed with saline, and split into two parts. One part was preserved in 10% formalin for microscopic examination, while the other was flash‐frozen in liquid nitrogen and kept at −80°C for later use.

#### 2.2.3 Insulin Plasma Measurements and Evaluation of HOMA‐IR, HOMA‐B, QUICKI, and DI

The concentration of insulin was determined through the use of an enzyme‐linked immunosorbent assay (ELISA) and a SunLong Biotech Co, China kit.

The following formulas were used to calculate homeostasis model assessment for insulin resistance (HOMAIR), homeostasis model assessment for β‐cell function (HOMA‐β), quantitative insulin sensitivity check index (QUICKI), and insulin sensitivity index (DI) [34]:

$$\text{HOMA}-\mathrm{IR}=\text{Insulin}\;\left(\mathrm{\mu IU}/\mathrm{mL}\right)\times \mathrm{FBG}\;\left(\mathrm{mg}/\mathrm{dL}\right)/405$$

$$\text{HOMA}-\beta =\text{Insulin}\;\left(\mathrm{\mu IU}/\mathrm{mL}\right)\times 360/\left[\mathrm{FBG}\;\left(\mathrm{mg}/\mathrm{dL}\right)-63\right]$$

$$\text{QUICKI}=1/\left[\log\;\mathrm{FBG}\;\left(\mathrm{mg}/\mathrm{dL}\right)+\log\;\text{insulin}\;\left(\mathrm{\mu IU}/\mathrm{mL}\right)\right]$$

InsulinDI:HOMA−β/HOMA−IR

#### 2.2.4 Determination of Lipid Profile

Plasma concentrations of triglycerides (TG), total cholesterol (TC), low‐density lipoprotein cholesterol (LDL‐c), and high‐density lipoprotein cholesterol (HDL‐c) were determined utilizing Pars Peyvand kits, Iran, with the autoanalyser technique.

The formulas for calculating the cholesterol ratio and atherogenic index (AI) were as follows:

Cholesterol ratio=TC/HDL−c

$$\mathrm{AI}=\log\;\left[\mathrm{TG}/\mathrm{HDL}-c\right]$$

#### 2.2.5 Real‐Time Polymerase Chain Reaction Analysis

Pancreatic tissues from all experimental groups underwent total RNA extraction via a commercial RNA extraction kit from Pars Tous, Iran, according to the manufacturer's protocol. RNA quantity and purity were assessed by spectrophotometry (NanoDrop, Thermo Scientific; S.N: D015). Complementary DNA (cDNA) was synthesized using the Easy cDNA synthesis kit (Pars Tous, Iran) following the provided instructions. A SYBR Green real‐time PCR kit from Pars Tous, Iran, was utilized on a Lava96 Real‐Time PCR system (DaAnGene Co. Ltd) to analyse gene expression of ACE1, AT1R, Keap‐1, and NAD(P)H: quinone oxidoreductase 1 (Nqo1), alongside miR‐200a and miR‐125b levels. To normalize mRNA and miRNA, GAPDH and U6 served as internal controls. Relative expression level calculations were performed via the ΔΔ Ct method [41]. Primer validation was performed before qPCR analysis. Amplification efficiency and linearity were assessed using efficiency plots, including correlation coefficient (*R* ^2^) analysis, and primer specificity was confirmed by melt‐curve analysis to verify reliable amplification of the target genes. Primer sequences (Pars Tous, Iran) are catalogued in Table 1.

**TABLE 1:** Real‐time PCR‐specific primers.

	Forward (5′‐3′)	Reverse (5′‐3′)
GAPDH	CATCACTGCCACCCAGAAGACTG	ATGCCAGTGAGCTTCCCGTTCAG
ACE1	TCAGCGGAATGGCGAAGTCCTA	CGGTCATACTCTTCCACGAACC
AT1R	GCCATTGTCCACCCGATGAAGT	ACACATTTCGGTGGATGACGGC
Keap‐1	ATCCAGAGAGGAATGAGTGGCG	TCAACTGGTCCTGCCCATCGTA
Nqo1	GCCGAACACAAGAAGCTGGAAG	GGCAAATCCTGCTACGAGCACT
U6	GCTCGCTTCGGCAGCACA	**—**
miR‐200a	TCTTACCGGACAGTGCTG	**—**
miR‐125b	CCCTGAGACCCTAACTTG	**—**

#### 2.2.6 Nrf2 and Phosphorylated NF‐кB P65 Analysed via Western Blot

The pancreatic samples were homogenized, and protein levels were subsequently measured using the Bradford procedure. After that, SDS‐PAGE was performed with equal amounts of protein samples, followed by transfer to nitrocellulose membranes. Antibodies targeting Nrf2, NFкB P65, and GAPDH (Santa Cruz Biotechnology; 1:1000, USA) were utilized for the identification of the relevant proteins. Protein bands were imaged by FUSIONFX Quantitative Imaging Device (Vilber, USA). For densitometric analysis, the optical density of each target protein band was quantified and normalized to the corresponding GAPDH band as the loading control. The relative expression levels of target proteins in the experimental groups were calculated by comparing the normalized target protein/GAPDH ratios with those obtained from the control group. Data were expressed as relative fold changes compared with the control group. Although each experimental group consisted of six animals, Western blot analysis was performed on protein samples obtained from three randomly selected animals per group (*n* = 3 independent biological replicates). Each replicate represented a distinct biological sample obtained from an individual experimental subject, rather than technical replicates of the same sample. The original uncropped Western blot images for Nrf2, NF‐κB, and GAPDH are provided in Files S1–, S3.

#### 2.2.7 Measurement of Pancreatic TNF‐α, IL‐1β and IL‐6 Levels

TNF‐α, IL‐1β, and IL‐6 levels were quantified in homogenized mouse pancreatic tissue with ELISA kits supplied by SunLong Biotech Co, China, following the procedures outlined in the manufacturer's guidelines.

#### 2.2.8 Assessment of Antioxidant Status

Pancreatic tissues were blended in chilled phosphate‐buffered saline that included a mixture of protease inhibitors, at a pH of approximately 7.4. The mixture was subsequently centrifuged at 6000 rpm for 10 min at 4°C. The supernatant obtained was employed to assess the concentrations of malondialdehyde (MDA) and total antioxidant capacity (TAC) using colorimetric techniques, in accordance with the instructions outlined in the Kiazist kits as indicated by the manufacturer.

#### 2.2.9 Histological Analysis

The pancreatic tissues were maintained in a 10% formalin solution; after that, they were embedded in paraffin and cut into sections of about 4–6 μm thickness. Following this setup, the sections were coloured using the haematoxylin and eosin (H&E) staining method. Histopathological examination was performed on tissue sections obtained from six animals per group (*n* = 6). The slides were evaluated independently by a pathologist blinded to the experimental groups initially. BMZ‐04‐DZ, a digital research microscope from Behin Pajouhesh ENG, was used to inspect the coloured tissue samples, and photographs were captured at a magnification of 200×. Representative photomicrographs are presented in the figures, whereas the quantitative histopathological scores were used for statistical analysis. Haemorrhage and congestion were assessed using a modified semi‐quantitative scoring system based on lesion severity: 0 = absent, 1 = minimal, 2 = mild, 3 = moderate, and 4 = severe.

### 2.3 Statistical Analysis

The results were subjected to statistical analysis utilizing the Statistical Package for Social Sciences (SPSS) software, version 25. This included one‐way ANOVA followed by Tukey's multiple comparisons test. The statistical outputs reported throughout the Results (*p*‐values, significance symbols in all figures) are indeed consistent with Tukey's test. Graphs were generated using GraphPad Prism software (version 9). All results are expressed as mean ± standard error (SE). Significance was set at *p* < 0.05 for differences.

## 3 Results

### 3.1 Effect of Harmaline on Secretion of Insulin by Isolated Mouse Islets

**FIGURE 1:**
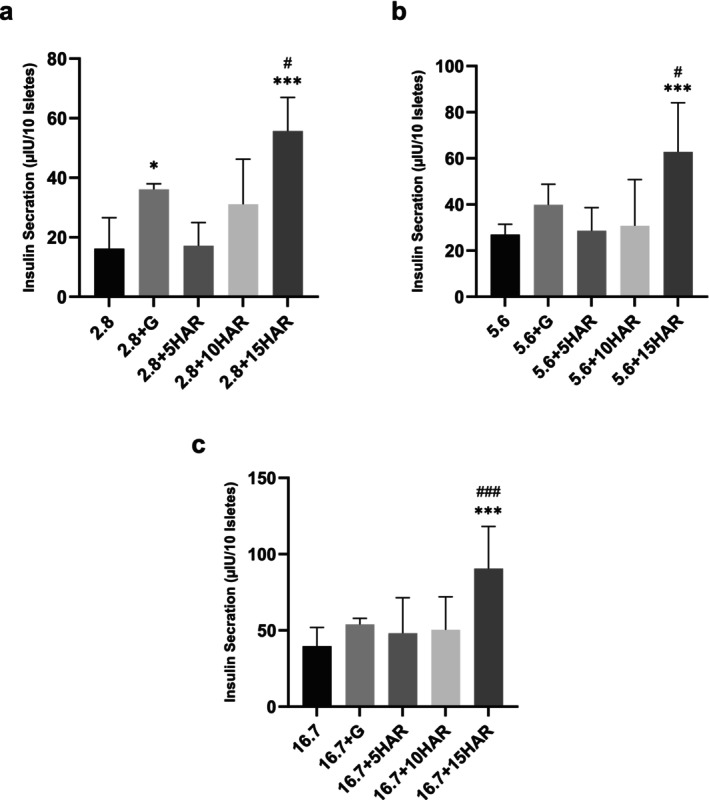
Effect of Harmaline on insulin secretion in media with different glucose concentrations in vitro: Insulin secretion in culture medium with (a) 2.8 mM glucose, (b) 5.6 mM glucose, (c) 16.7 mM glucose. Data are presented as mean ± SEM. *Compared to the control group and # compared to the glibenclamide group. 1, 2, and 3 symbols indicate statistically significant differences with *p* < 0.05, *p* < 0.01, and *p* < 0.001, respectively.

Male mouse islet insulin secretion was assessed in media containing 2.8, 5.6, and 16.7 mM glucose. In the culture medium with 2.8 mM glucose, both 10 μM glibenclamide and 15 μM harmaline significantly increased insulin secretion compared with the control group. Moreover, insulin release was considerably greater in the harmaline‐treated islets than in the glibenclamide‐treated group, while the remaining groups showed no significant differences (Figure 1a). In the culture medium with 5.6 and 16.7 mM glucose, 15 μM harmaline markedly enhanced insulin secretion compared with both the control and glibenclamide groups (Figure 1b,c).

### 3.2 Effect of Harmaline on Lipid Peroxidation and Antioxidant Capacity of Islets of Langerhans Isolated From Male Mice

**FIGURE 2:**
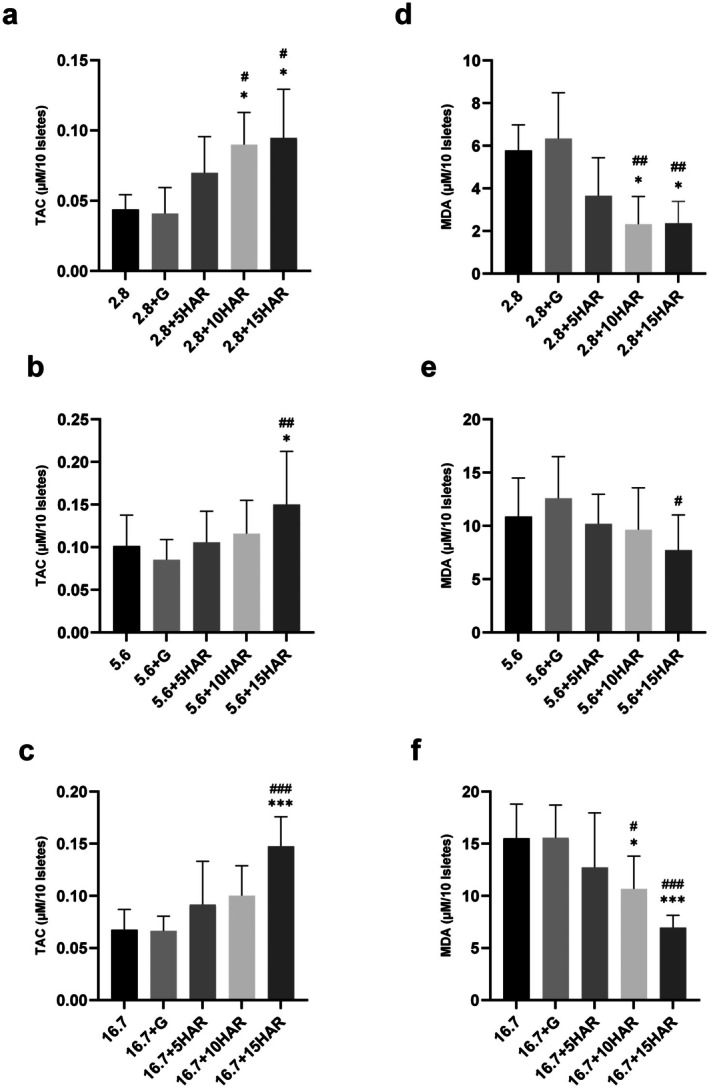
Effect of Harmaline on TAC and MDA in media with different glucose concentrations in vitro: TAC in culture medium with (a) 2.8 mM glucose, (b) 5.6 mM glucose, (c) 16.7 mM glucose and MDA in culture medium with (d) 2.8 mM glucose, (e) 5.6 mM glucose, (f) 16.7 mM glucose. Data are presented as mean ± SEM. *Compared to the control group and # compared to the glibenclamide group. 1, 2, and 3 symbols indicate statistically significant differences with *p* < 0.05, *p* < 0.01, and *p* < 0.001, respectively.

In Figure 2a, at a glucose concentration of 2.8 mM, treatment of islets with HAR at doses of 10 and 15 μM rise in TAC versus the untreated control group and the glibenclamide group (as a positive control group). As displayed in Figure 2d, MDA, a reliable marker of lipid peroxidation, did not show a significant difference between 5 harmaline and glibenclamide, in contrast to the untreated control group. Be that as it may, treatment with 10 μM and 15 μM harmaline notably decreased MDA compared to the untreated glibenclamide.

Figure 2b,e illustrate how HAR and glibenclamide affect TAC and MDA in islets with 5.6 mM glucose. There was no noteworthy contrast in TAC and MDA between the untreated control and glibenclamide groups. Treatment with 15 μM harmaline significantly increased TAC compared to the untreated control and glibenclamide groups. MDA content was considerably diminished in the 15 μM harmaline groups in comparison with the glibenclamide group.

In examining the effects of HAR at a glucose concentration of 16.7 mM, in Figure 2c, treatment of islets with HAR at a dose of 15 μM significantly increased TAC compared to untreated control and glibenclamide groups. However, MDA content in the 10 and 15 μM harmaline groups demonstrated a substantially reduced level relative to the untreated control and glibenclamide groups. In Figure 2f, this diminution was more noticeable in the 15 μM harmaline group.

### 3.3 Effect of Harmaline on Glycemic Control, Insulin Resistance, and β‐Cell Function in T2DM Mice

**FIGURE 3:**
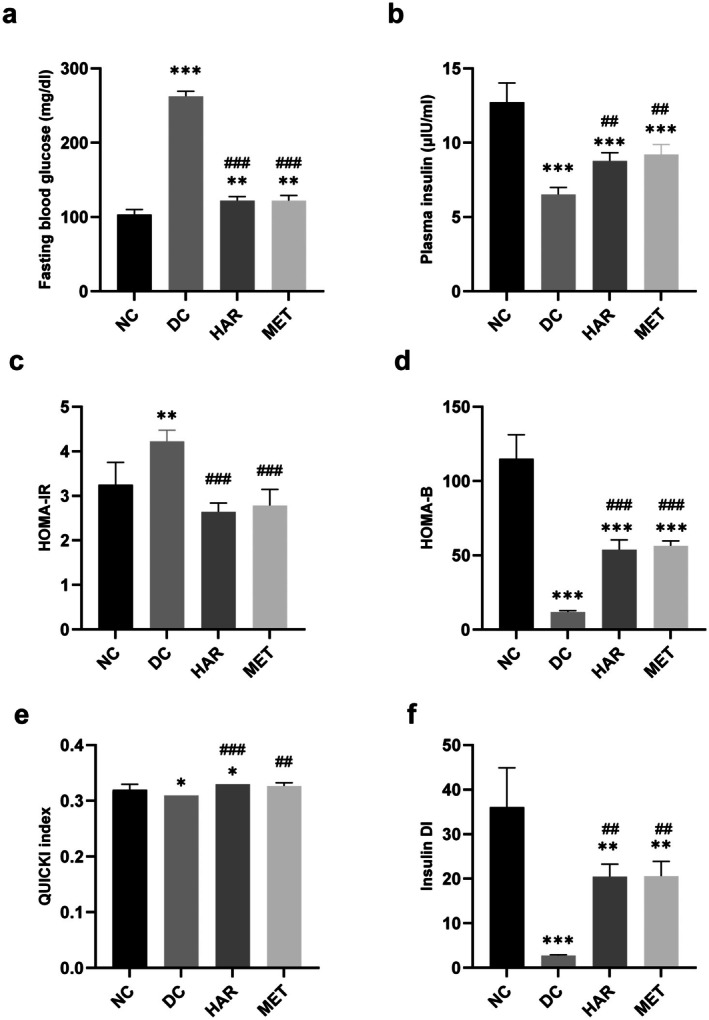
Effects of HAR on: (a) fasting blood glucose, (b) plasma insulin, (c) HOMA‐IR, homeostatic model assessment for insulin resistance, (d) HOMA‐B, homeostasis model assessment of β‐cell function, (e) QUICKI, quantitative insulin sensitivity check index, and (f) DC, diabetic control group; DI, insulin disposition index; HAR, test group receiving Harmaline; MET, metformin positive control group; NC, normal control group and data are represented in mean ± SEM, *n* = 6 in each group. *Compared to NC group and # compared to DC group. $ was used for comparison of MET; positive control and HAR groups. $ was used for comparison of MET; positive control and HAR groups. 1, 2, and 3 symbols represent statistically significant differences with *p* < 0.05, *p* < 0.01, and *p* < 0.001, respectively.

Type 2 diabetes was induced by giving NA and STZ. The diabetic mice showed significantly elevated FBG 7 days post‐induction, validating the diabetic model (Figure 3a). Treatment with harmaline or metformin significantly reduced FBG levels relative to diabetic controls.

Plasma insulin levels were markedly decreased in diabetic mice, whereas both harmaline and metformin significantly restored insulin concentrations (Figure 3b). Diabetic mice also exhibited significantly higher HOMA‐IR values and lower HOMA‐B, QUICKI, and disposition index (DI) values compared with normal controls, showing insulin resistance, poor insulin sensitivity, and malfunctioning β‐cells (Figure 3c–f). Treatment with harmaline or metformin significantly decreased HOMA‐IR and increased HOMA‐B, QUICKI, and DI indices compared with diabetic controls, reflecting improved insulin sensitivity and β‐cell function.

### 3.4 Effect of Harmaline on the Plasma Lipid Profile in T2DM Mice

**FIGURE 4:**
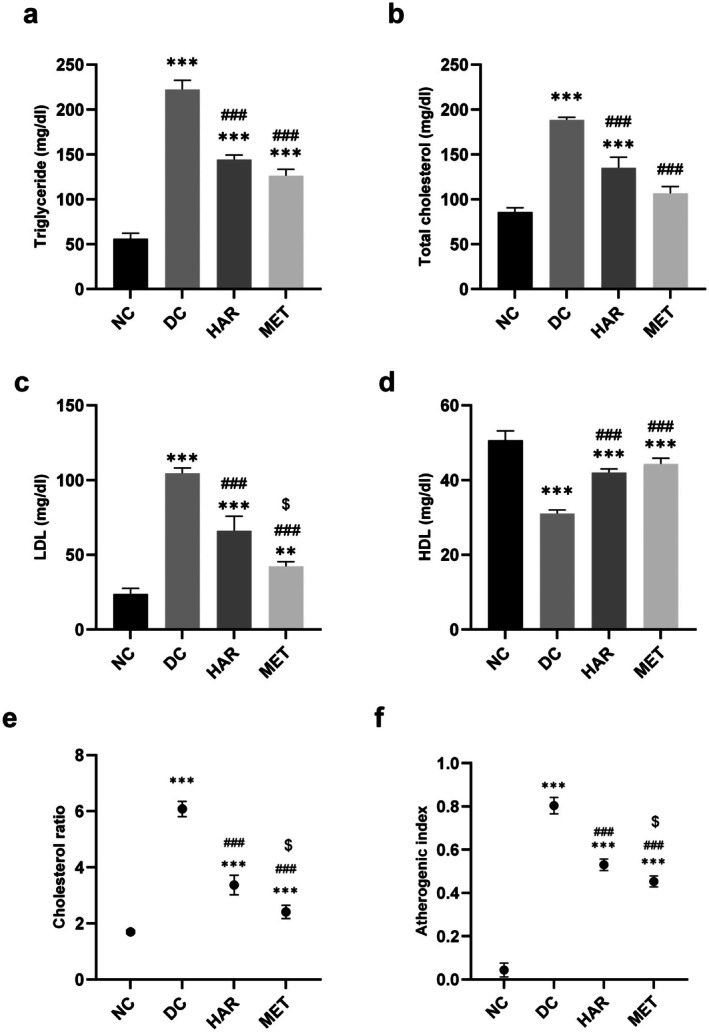
Effects of HAR on: (a) TG, triglyceride, (b) T‐CHO, total cholesterol, (c) LDL, low‐density lipoprotein, (d) HDL, high‐density lipoprotein of mice plasma, (e) cholesterol ratio, and (f) Atherogenic index. DC, diabetic control group; HAR, test group receiving Harmaline; MET, metformin positive control group; NC, normal control group. Data are represented in mean ± SEM, *n* = 6 in each group. *Compared to NC group and # compared to DC group. $ was used for comparison of MET; positive control and HAR groups. $ was used for comparison of MET; positive control and HAR groups. 1, 2, and 3 symbols represent statistically significant differences with *p* < 0.05, *p* < 0.01, and *p* < 0.001, respectively.

The diabetic control group exhibited significantly elevated TG and TC levels compared with normal controls (Figure 4a,b). HAR or metformin treatment led to a significant decrease in levels of TG and TC. Similarly, diabetic mice exhibited elevated LDL‐C levels, but HAR and metformin decreased LDL‐C compared with them (Figure 4c). HDL‐C levels were notably increased following HAR treatment, comparable to the effect of metformin (Figure 4d). Furthermore, the cholesterol ratio and atherogenic index, which were elevated in diabetic mice, were significantly lowered by HAR and metformin (Figure 4e,f), suggesting reduced cardiovascular risk.

### 3.5 Effect of Harmaline on Oxidative Stress Markers and Antioxidant Capacity in Pancreatic Tissue

**FIGURE 5:**
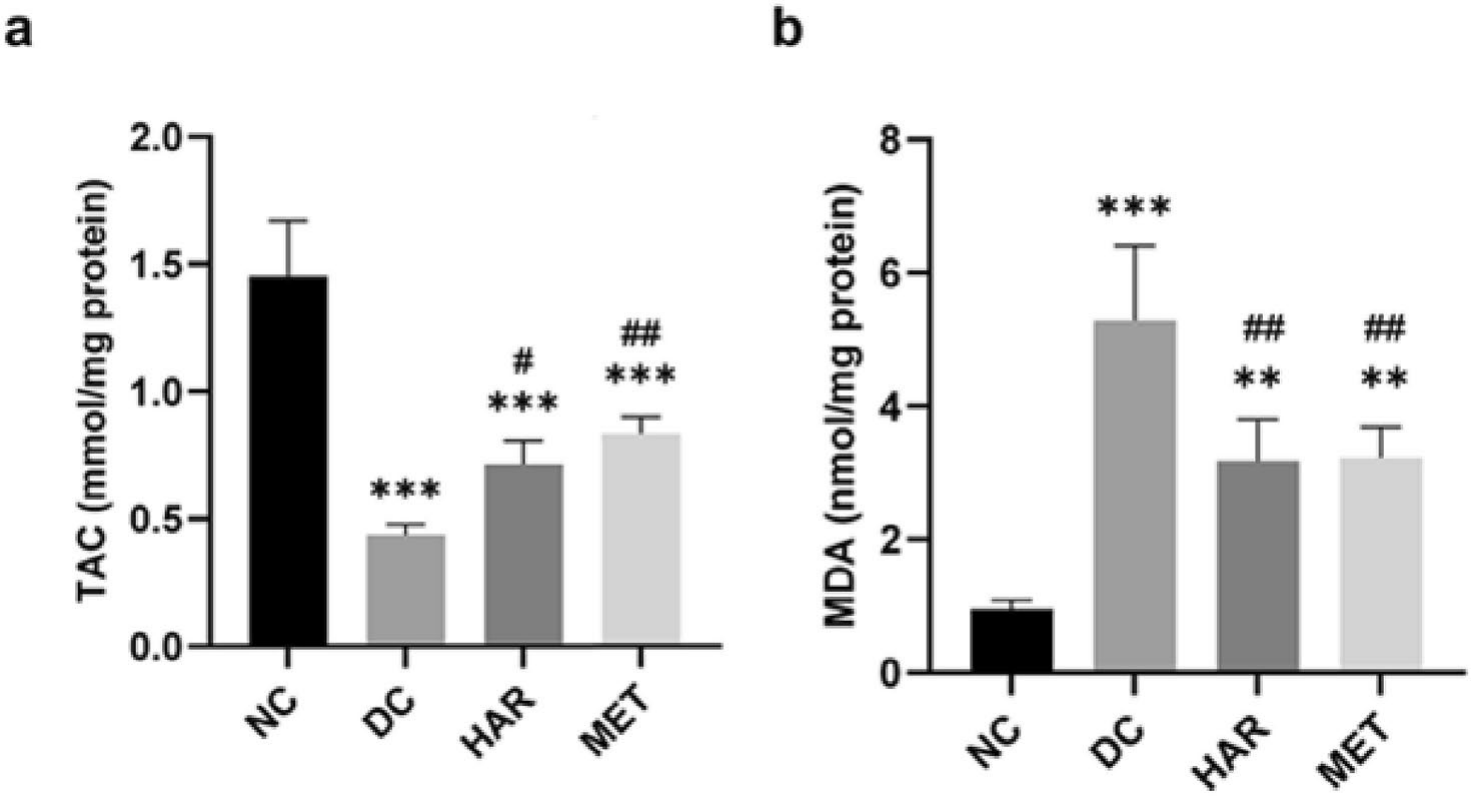
Effects of HAR on: (a) TAC, total antioxidant capacity, and (b) DC, diabetic control group; MDA, malondialdehyde; NC, normal control group; HAR, test group receiving Harmaline, and MET, metformin positive control group. Data are represented in mean ± SEM, *n* = 6 in each group. *Compared to NC group and # compared to DC group. $ was used for comparison of MET; positive control and HAR groups. $ was used for comparison of MET; positive control and HAR groups. 1, 2, and 3 symbols represent statistically significant differences with *p* < 0.05, *p* < 0.01, and *p* < 0.001, respectively.

Oxidative stress markers, including TAC and MDA, were assessed in pancreatic tissue (Figure 5a,b). The diabetic control group showed a notable decline in TAC levels relative to the normal control group, pointing to a compromised antioxidant defence system (Figure 5a). TAC levels were notably boosted by treatment with HAR and metformin. Conversely, MDA levels were markedly elevated in diabetic mice, reflecting enhanced oxidative stress (Figure 5b). Both HAR and metformin significantly reduced MDA levels compared with the diabetic control group, demonstrating their antioxidant potential.

### 3.6 Effect of Harmaline on the Nrf2 Pathway, Keap1, NQO1, and miR‐200a Expression in Pancreatic Tissue

**FIGURE 6:**
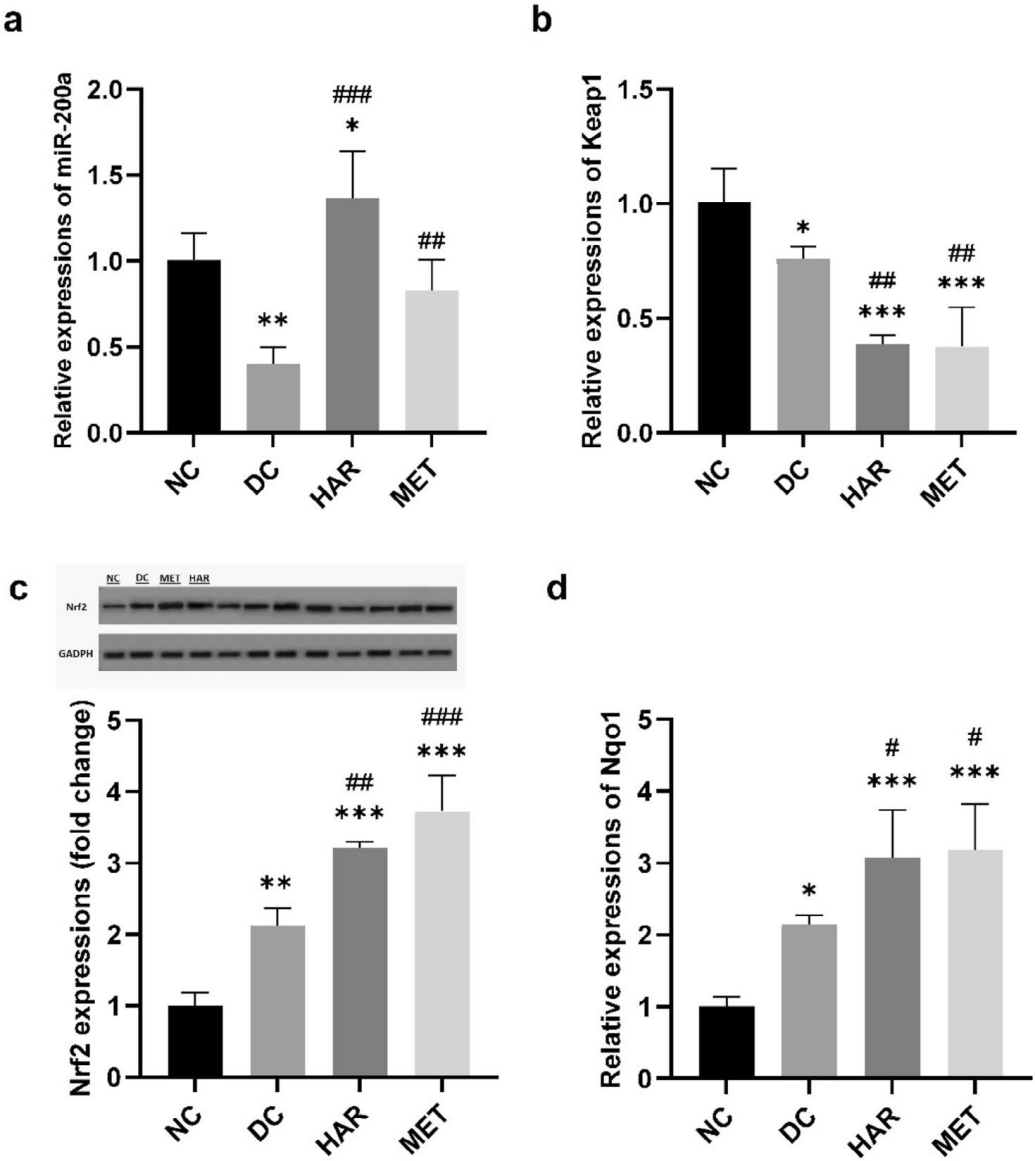
Effects of HAR on: (a) miR‐200a, (b) Keap1, (c) Nrf2 (western blot analysis), and (d) Nqo1 relative expression in the pancreatic tissue. DC, diabetic control group; HAR, test group receiving Harmaline; MET, metformin positive control group; NC, mormal control group. Data are represented in mean ± SEM, *n* = 6 in each group for mRNAs and *n* = 3 in each group for western blot. The lanes in Western Bolt, from left to right, represent the groups of NC, DC, MET, and HAR, respectively. This arrangement was repeated three times. *Compared to NC group and # compared to DC group. $ was used for comparison of MET; positive control and HAR groups. $ was used for comparison of MET; positive control and HAR groups. 1, 2, and 3 symbols represent statistically significant differences with *p* < 0.05, *p* < 0.01, and *p* < 0.001, respectively.

The effects of HAR on the Nrf2 signalling pathway were investigated in pancreatic tissue using Western blotting and qRT‐PCR (Figure 6a–d). In diabetic mice, miR‐200a levels were significantly reduced compared to healthy mice, while HAR and metformin treatment markedly upregulated miR‐200a levels (Figure 6a). Compared to control groups, diabetic mice had lower Keap1 expression, with a further drop in HAR‐ and metformin‐treated groups (Figure 6b). Nrf2 protein levels were elevated in diabetic mice and showed a further significant increase following HAR administration, which was also observed with metformin treatment (Figure 6c). This enhancement in Nrf2 expression is likely associated with the reduction of Keap1 in the treated groups. Consistently, Nqo1 expression was upregulated in diabetic mice and exhibited additional increases after HAR and metformin treatment (Figure 6d), confirming the activation of the Nrf2 antioxidant pathway.

### 3.7 Effect of Harmaline at the Expression of miR‐125b and NF‐кB P65, in Addition to the Concentrations of TNF‐α, IL‐1ß, and IL‐6 in Pancreatic Tissue

**FIGURE 7:**
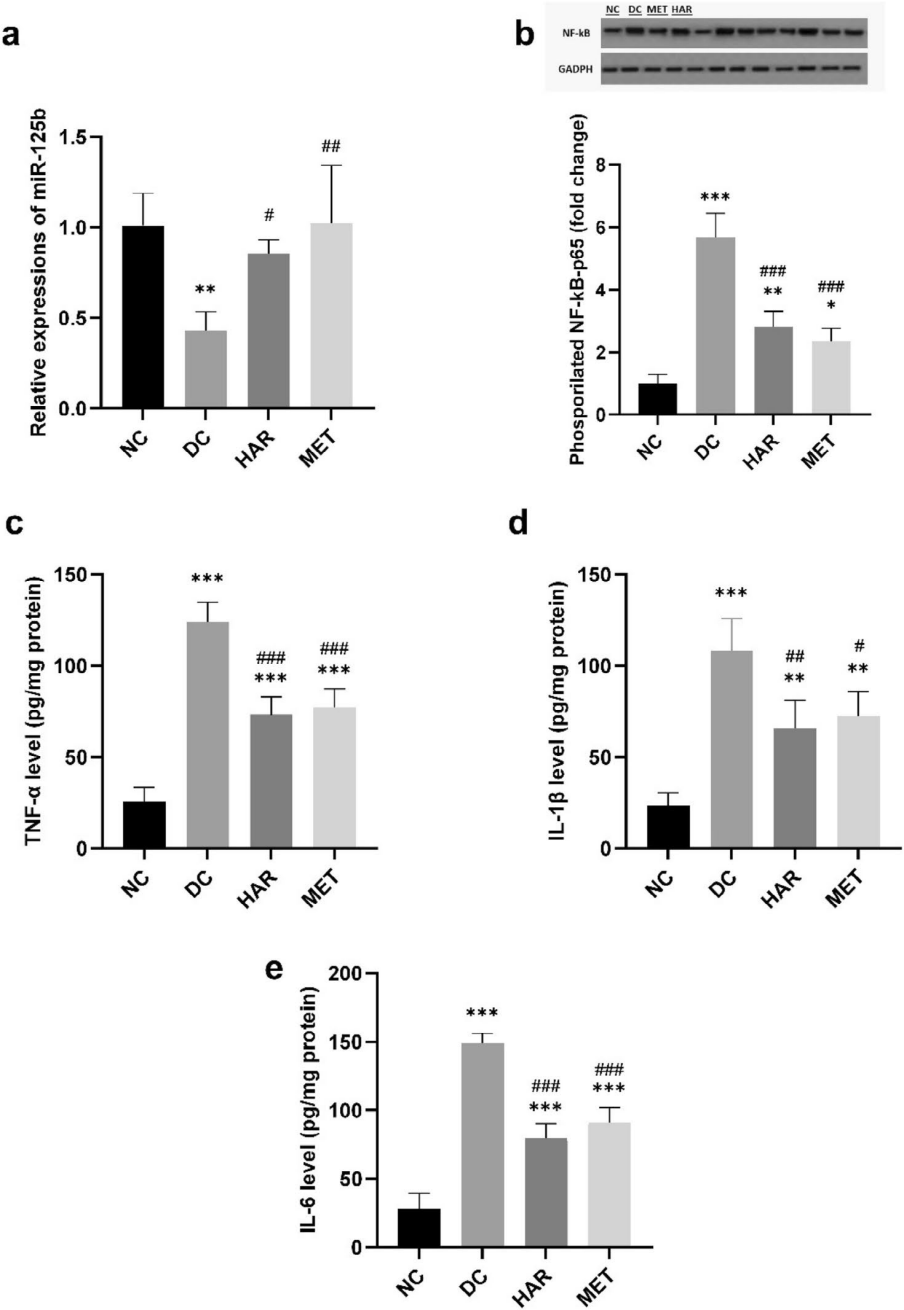
Effects of HAR on: (a) miR‐125b relative expression, (b) NF‐kB‐P65 relative expression (western blot analysis), (c) TNF‐α, (d) IL‐1ß, and (e) IL‐6 concentration of mice pancreatic tissue. DC, diabetic control group; HAR, test group receiving Harmaline; MET, metformin positive control group; NC, normal control group. Data are represented in mean ± SEM, *n* = 6 in each group, and *n* = 3 in each group for the western blot. The lanes in Western Bolt, from left to right, represent the groups of NC, DC, MET, and HAR, respectively. This arrangement was repeated three times. *Compared to NC group and # compared to DC group. $ was used for comparison of MET; positive control and HAR groups. $ was used for comparison of MET; positive control and HAR groups. 1, 2, and 3 symbols represent statistically significant differences with *p* < 0.05, *p* < 0.01, and *p* < 0.001, respectively.

The HAR's effect on inflammatory signalling was evaluated by measuring miR‐125b expression and inflammatory markers in pancreatic tissue. Compared to normal controls, diabetic mice had a significantly decreased miR‐125b expression; in contrast, HAR or metformin treatment substantially elevated expression (Figure 7a). A significant increase in NF‐κB p65 levels was also observed due to diabetes induction, while HAR and metformin treatment effectively suppressed its expression (Figure 7b). Furthermore, significantly more pro‐inflammatory cytokines (TNF‐α, IL‐1β, and IL‐6) were found in the diabetic control group than in normal mice (Figure 7c–e). Administration of HAR and metformin considerably diminished the presence of these inflammatory markers relative to the diabetic control group, indicating attenuation of diabetes‐associated inflammatory responses.

### 3.8 Effects of Harmaline on RAS in Pancreatic Tissue

**FIGURE 8:**
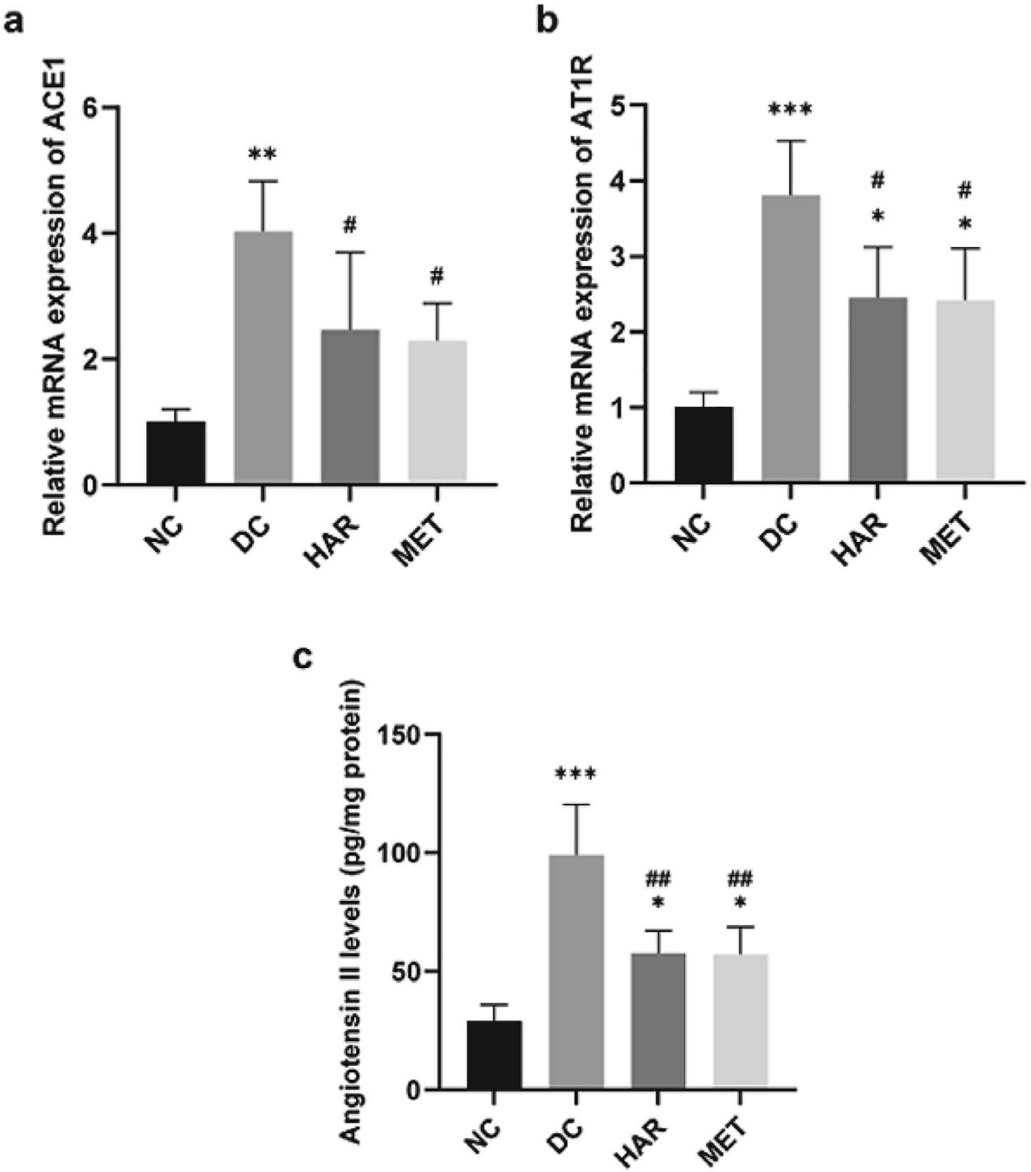
Effects of HAR on: (a) ACE1 relative expressions, (b) AT1R expressions, (c) Angiotensin II concentration of mice pancreatic tissue. DC, diabetic control group; HAR, test group receiving Harmaline; MET, metformin positive control group; NC, normal control group. Data are represented in mean ± SEM, *n* = 6 in each group. *Compared to NC group and # compared to DC group. $ was used for comparison of MET; positive control and HAR groups. $ was used for comparison of MET; positive control and HAR groups. 1, 2, and 3 symbols represent statistically significant differences with *p* < 0.05, *p* < 0.01, and *p* < 0.001, respectively.

The role of localized RAS in the pancreas as a factor influencing cell growth and glucose‐stimulated insulin release from islets of Langerhans was evaluated. Compared with normal controls, ACE1, AT1R expression, and angiotensin II (Ang II) levels were substantially elevated in diabetic mice. A reduction was seen with HAR and metformin treatment in these three factors (Figure 8a–c). These findings suggest that HAR may exert protective effects in diabetes by suppressing the overactivation of the pancreatic RAS pathway.

### 3.9 The Impact of HAR on the Histopathology Examination of the Pancreas

**FIGURE 9:**
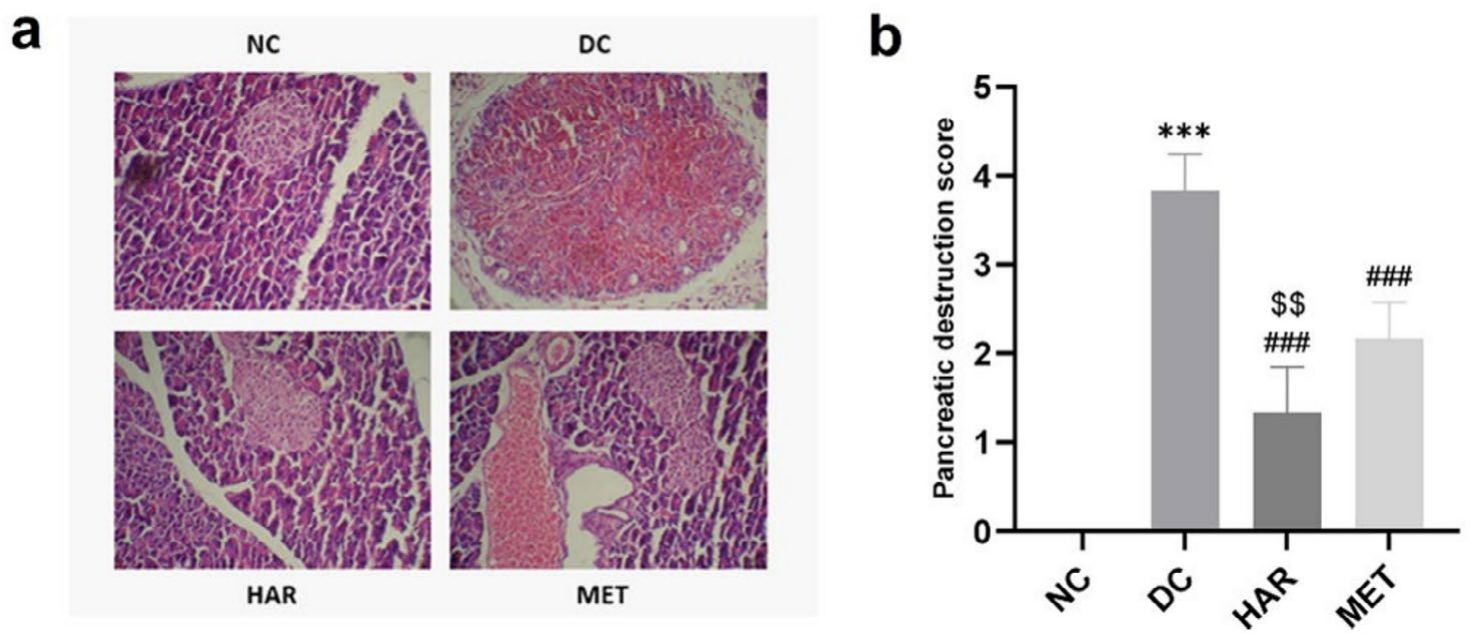
(a) Effects of HAR and Metformin on histology of the pancreas in NA‐STZ‐induced type 2 diabetes mellitus. (b) Semi‐quantitative histopathological scores for haemorrhage and congestion (0–4) are presented as mean ± SD (*n* = 6 animals per group). DC, diabetic control group; HAR, test group receiving Harmaline; MET, metformin positive control group; NC, normal control group. Representative samples are shown with H&E staining. *Compared to NC group and # compared to DC group. $ was used for comparison of MET; positive control and HAR groups. 1, 2, and 3 symbols represent statistically significant differences with *p* < 0.05, *p* < 0.01, and *p* < 0.001, respectively.

Histopathological evaluation was performed to examine pancreatic damage in T2DM by haematoxylin and eosin staining. To achieve this goal in the pancreas, changes in haemorrhage and congestion were evaluated. Figure 9a indicates that there was no haemorrhage or congestion in the control samples, whereas pancreatic haemorrhage and congestion were observed in the diabetic group. Bleeding was mild in the metformin group, but congestion persisted. In the HAR group, mild bleeding and congestion were observed. Semi‐quantitative histopathological scoring (Figure 9b) demonstrated significant differences in pancreatic haemorrhage and congestion among the experimental groups. The diabetes group exhibited significantly more severe haemorrhage and congestion scores compared with the control group. Treatment with HAR and Metformin significantly reduced both haemorrhage and congestion compared with the diabetic group. The reduction in bleeding and congestion was reported to be minimal in the harmaline‐treated group and mild in the metformin‐treated group.

## 4 Discussion

Given the increasing prevalence of T2DM worldwide, identifying effective therapeutic approaches with fewer adverse effects remains important. In the in vitro part of the present study, mouse pancreatic islets were cultured under different glucose concentrations to evaluate the effects of HAR on insulin secretion and oxidative stress markers. Previous studies have demonstrated that antioxidants can alleviate high‐glucose‐induced β‐cell dysfunction, support cell survival, and improve insulin secretion [42]. Similarly, antioxidant supplementation in pancreatic β‐cell culture under high glucose conditions has been shown to reduce ROS production [43]. Consistent with these findings, the present study showed that treatment with 15 μM HAR under different glucose concentrations enhanced insulin secretion, increased total antioxidant capacity, and reduced malondialdehyde levels. These results suggest that HAR may improve β‐cell function under diabetic‐like conditions, potentially through modulation of glucose‐induced oxidative stress. The in vitro findings further support the observations obtained from the in vivo experiments.

It is essential to acknowledge that the development and advancement of T2DM are critically dependent on oxidative stress and inflammation. For further review in this research, we explored the effect of HAR in T2DM mice, the NA‐STZ model. Results from the present study indicated that HAR led to a considerable lowering of blood glucose and enhanced insulin concentrations, contributing to better insulin responsiveness and glycemic indices. The NA‐STZ‐induced diabetic model has also been used in our previous work [34], although the therapeutic agent investigated in the present study was different. These observations align with prior evidence that an antioxidant improved glycemic indices, comprising HOMA‐IR, HOMA‐B, QUICKI, and DI [44]. Insulin regulates lipid metabolism; its deficiency leads to an increase in acyl‐CoA, which provides a substrate for the β‐oxidation of free fatty acids, generates free acetate, and upregulates liver cholesterol biosynthesis [45]. These findings are similar to a study by Negmatova et al., which found that dyslipidemia occurs with elevated LDL‐C, TC, and TG in the blood and lower HDL‐C in the serum of individuals diagnosed with T2DM [46]. Also, the outcomes of this study showed that HAR remarkably lowers TG, TC, and LDL‐C levels while increasing HDL‐C levels. This observation supports earlier evidence indicating that, in the study of Berrougui et al., harmaline inhibited the aggregation and oxidation of LDL protein [47].

Activation of the Nrf2 antioxidant defence pathway has been recognized as a potential therapeutic strategy for mitigating hyperglycemia‐associated oxidative damage and diabetic complications [48]. In the present study, administration of HAR increased Nrf2 protein expression compared with untreated diabetic mice, which is consistent with the proposed antioxidant properties of this compound and its potential involvement in Nrf2 pathway activation. Previous studies have demonstrated that miR‐200a can negatively regulate Keap1 expression, thereby facilitating Nrf2 activation [49, 50]. However, unlike previous findings in diabetic nephropathy, where reduced miR‐200a expression was associated with increased Keap1 expression [51], the present study observed a simultaneous reduction in miR‐200a and Keap1 mRNA levels in the pancreas of type 2 diabetic mice. This finding suggests that regulation of Keap1 in diabetic pancreatic tissue may involve additional mechanisms beyond miR‐200a‐mediated regulation. The decreased miR‐200a expression observed in diabetic mice may be related to diabetes‐associated alterations, including oxidative stress, inflammation, and β‐cell dysfunction. Nevertheless, the current study was not designed to identify the upstream regulators of miR‐200a or to establish direct regulatory interactions within the miR‐200a/Keap1 axis. The observed increase in oxidative stress markers (elevated MDA and reduced TAC) and inflammatory responses in diabetic mice may represent additional factors influencing Keap1/Nrf2 signalling. Indeed, previous studies have suggested that oxidative and inflammatory pathways can modulate Keap1 expression and Nrf2 activation through multiple regulatory mechanisms [52]. Therefore, the current findings indicate an association between alterations in miR‐200a, Keap1, and Nrf2 signalling during diabetic pancreatic injury; however, further investigations are required to clarify the molecular mechanisms underlying this regulatory network. Nqo1 is a well‐established downstream target gene of Nrf2 and contributes to cellular antioxidant defence through detoxification of quinones and regulation of redox homeostasis [53, 54, 55]. Moreover, Nqo1 has been reported to modulate antioxidant enzymes, including SOD and GPx, downstream of Nrf2 activation [56] and plays an important role in protection against oxidative stress [57]. In the present study, mice treated with HAR exhibited increased Nqo1 gene expression, which was accompanied by enhanced TAC levels and reduced MDA accumulation, further supporting the activation of antioxidant defence mechanisms.

Inflammation is a prominent feature of T2DM, with inflammatory processes occurring in the islets of Langerhans and contributing to β‐cell dysfunction and loss [58]. T2DM is associated with increased inflammatory markers and mediators, including IL‐6 and IL‐1β, in pancreatic islets. Elevated NOX activity in diabetic conditions may also contribute to increased TNF‐α levels [59]. Inflammatory mediators and chemokines can activate NF‐κB signalling, which is involved in pancreatic β‐cell impairment [60]. Accordingly, reducing NF‐κB, TNF‐α, IL‐6, and IL‐1β levels has been reported to improve hyperglycaemia, insulin secretion, and β‐cell function [61, 62]. Consistent with these findings, the present study showed increased NF‐κB expression and elevated TNF‐α, IL‐1β, and IL‐6 levels in diabetic mice, whereas these alterations were attenuated following treatment with HAR and metformin. The increased MDA levels, reduced TAC, and altered islet morphology observed in diabetic mice may be associated with enhanced oxidative stress and inflammatory responses, including activation of NF‐κB and increased pro‐inflammatory cytokine production. Moreover, miR‐125b has been implicated in the regulation of β‐cell function and insulin secretion, and its reduced expression has been associated with impaired β‐cell activity and insulin sensitivity [63]. Previous studies have also suggested that decreased miR‐125b expression may be linked to activation of NF‐κB signalling and inflammatory responses in liver injury models [64]. In agreement with these reports, the present study demonstrated that NA/STZ‐induced T2DM was associated with decreased miR‐125b expression and increased NF‐κB expression. Furthermore, increased miR‐125b expression and attenuation of NF‐κB elevation were observed in the groups treated with HAR and metformin.

ACE1 initiates physiological and pathological responses by producing Ang II and exerting Ang II effects on the AT1R receptor [65]. Diabetes has been shown to overexpress mRNA and protein AT1R [66], as well as to increase plasma Ang II levels [67]. Data suggest that increased RAS activity in the pancreas adversely impacts insulin secretion by reducing pancreatic blood flow, inducing oxidative stress, inflammation, and fibrosis of the islets of Langerhans. Also, skeletal muscle and adipose tissue dysfunction may contribute to RAS‐induced insulin resistance [19]. Prevention of T2DM by RAS inhibition may be due to improved beta‐cell function and/or increased insulin sensitivity [68]. These studies corroborated the current finding, which emphasized that the expression of ACE1, AT1R, and Ang II rose in mice with diabetes, and treatment with HAR and metformin in diabetic mice showed improvement in components of the RAS system.

Medicinal plants with antioxidant properties are known to manage diabetes complications and control metabolism [69]. These findings indicate that harmaline helps to improve the status and disorders of T2DM both in vitro and in vivo. The protective measures of harmaline are partly due to its ability to repair tissue damage, as well as to increase antioxidant activity and reduce inflammation through various pathways. These findings align with other research indicating harmaline's antioxidant and anti‐inflammatory properties [28, 70]. Given that harmaline is effective in reducing oxidative stress and inflammation, it is reasonable to suggest that this compound, which is derived from the plant *Peganum harmala* , may have a beneficial role in the management of T2DM.

This study has limitations that should be considered when interpreting the findings. HAR showed beneficial effects in this experimental model; further studies using multiple dose levels, longer treatment durations, and detailed toxicological evaluations are required to confirm its safety profile and therapeutic potential. A limitation of this study is the relatively small number of biological replicates used for Western blot analysis (*n* = 3 per group). However, the observed protein‐level changes were supported by complementary qPCR analyses of related pathway‐associated targets, which provided additional molecular evidence consistent with the Western blot findings. Future studies with larger sample sizes and additional validation approaches will further strengthen these observations. The exclusive use of male mice represents a limitation of the present study. Male mice were selected to minimize variability associated with hormonal fluctuations and to establish a controlled experimental model for this initial investigation.

## 5 Conclusion

The present study provides evidence that HAR exerts protective effects in an NA–STZ‐induced T2DM model. HAR administration enhanced insulin secretion, improved the lipid profile, restored pancreatic antioxidant capacity, reduced FBG levels, and attenuated inflammatory responses. These beneficial effects were associated with increased Nrf2 expression, reduced NF‐κB expression, and modulation of RAS‐related markers, suggesting a potential involvement of these pathways in the observed protective effects. In diabetic mice with pancreatic injury and disease progression, dysregulated expression of miR‐200a and miR‐125b was observed, indicating that these microRNAs may contribute to β‐cell dysfunction and diabetes progression. Collectively, these findings suggest that HAR may represent a promising candidate for improving glycemic control and reducing pancreatic oxidative stress and inflammation in T2DM. However, further mechanistic studies are needed to establish causal relationships between these molecular changes and the observed protective effects.

## Author Contributions

**Farima Malekinia:** data curation, formal analysis, methodology, writing – original draft. **Akram Ahangarpour:** data curation, methodology, funding acquisition, formal analysis, investigation, supervision, validation, writing – review and editing, resources, project administration, conceptualization. **Seyyed Ali Mard:** writing – review and editing, formal analysis, visualization. **Khojasteh Hoseinynejad:** writing – review and editing, visualization, validation. **Maryam Radan:** writing – review and editing, software, methodology. **Fereshteh Nejaddehbashi:** writing – review and editing, validation, methodology.

## Funding

This work was supported by Ahvaz Jundishapur University of Medical Sciences, D‐0304.

## Conflicts of Interest

The authors declare no conflicts of interest.

## Supporting information


**File S1:** edm270304‐sup‐0001‐FileS1.tif.

**File S2:** edm270304‐sup‐0002‐FileS2.tif.

**File S3:** edm270304‐sup‐0003‐FileS3.tif.

## Data Availability

The data that support the findings of this study are available on request from the corresponding author.
